# Supplementary material for: The role of neighbourhood socioeconomic status in large for gestational age
Source: PLoS One. 2020 Jun 5;15(6):e0233416. doi: 10.1371/journal.pone.0233416 (PMC7274403; doi:10.1371/journal.pone.0233416)
Supplement: S1 Table — (DOCX) [file pone.0233416.s001.docx]

Supplementary table 1. Comparison of the neighborhood deprivation index used in the study with related variables from the 2015 French National Census data

| **2015 Census data in the city of Marseilles** | **Neighborhood Deprivation Index** | | | |
| --- | --- | --- | --- | --- |
|  | **Q1** | **Q2** | **Q3** | **Q4** |
| **Mean household income (Euros)** | 23,479 | 17,305 | 13,816 | 6604 |
| **Portion of the population aged 15 years and older with a high school degree (%)** | 54 | 42 | 35.5 | 23 |
| **Unemployment rate (%)** | 12.7 | 16.05 | 24.1 | 29.1 |
| **Blue-collar workers (%)** | 39.4 | 53 | 57.5 | 71.3 |
