# Supplementary material for: The role of neighbourhood socioeconomic status in large for gestational age
Source: PLoS One. 2020 Jun 5;15(6):e0233416. doi: 10.1371/journal.pone.0233416 (PMC7274403; doi:10.1371/journal.pone.0233416)
Supplement: Attachment — Submitted filename: Response to reviewers_Letter_17032020.docx [file pone.0233416.s003.docx]

Editor

| Editor comments | **Author response and changes made** | **Page number** |
| --- | --- | --- |
| 1. Please ensure that your manuscript meets PLOS ONE's style requirements, including those for file naming. The PLOS ONE style templates can be found at [http://www.plosone.org/attachments/PLOSOne_formatting_sample_main_body.pdf](https://urldefense.com/v3/__http:/www.plosone.org/attachments/PLOSOne_formatting_sample_main_body.pdf__;!!JQ5agg!J26tD8jOp8XofgXZtoLkz1ia9DJnQaKXKBap8EponUvoq88SIvatW3s-tG53KtKS1frq-A$) and [http://www.plosone.org/attachments/PLOSOne_formatting_sample_title_authors_affiliations.pdf](https://urldefense.com/v3/__http:/www.plosone.org/attachments/PLOSOne_formatting_sample_title_authors_affiliations.pdf__;!!JQ5agg!J26tD8jOp8XofgXZtoLkz1ia9DJnQaKXKBap8EponUvoq88SIvatW3s-tG53KtLTBoxqsQ$) | We checked the manuscript in accordance with PLOS ONE's style requirements | Not applicable |
| 2. Please provide additional details regarding participant consent. In the ethics statement in the Methods and online submission information, please ensure that you have specified (1) whether consent was informed and (2) what type you obtained (for instance, written or verbal). If your study included minors, state whether you obtained consent from parents or guardians. If the need for consent was waived by the ethics committee, please include this information. | No consent, medico-administrative data, all details are present in the ethics section | Page 6 |
| 3. We note that you have indicated that data from this study are available upon request. PLOS only allows data to be available upon request if there are legal or ethical restrictions on sharing data publicly. For information on unacceptable data access restrictions, please see [http://journals.plos.org/plosone/s/data-availability#loc-unacceptable-data-access-restrictions](https://urldefense.com/v3/__http:/journals.plos.org/plosone/s/data-availability*loc-unacceptable-data-access-restrictions__;Iw!!JQ5agg!J26tD8jOp8XofgXZtoLkz1ia9DJnQaKXKBap8EponUvoq88SIvatW3s-tG53KtLAmo7eUg$).    In your revised cover letter, please address the following prompts:    a) If there are ethical or legal restrictions on sharing a de-identified data set, please explain them in detail (e.g., data contain potentially identifying or sensitive patient information) and who has imposed them (e.g., an ethics committee). Please also provide contact information for a data access committee, ethics committee, or other institutional body to which data requests may be sent.    b) If there are no restrictions, please upload the minimal anonymized data set necessary to replicate your study findings as either Supporting Information files or to a stable, public repository and provide us with the relevant URLs, DOIs, or accession numbers. Please see [http://www.bmj.com/content/340/bmj.c181.long](https://urldefense.com/v3/__http:/www.bmj.com/content/340/bmj.c181.long__;!!JQ5agg!J26tD8jOp8XofgXZtoLkz1ia9DJnQaKXKBap8EponUvoq88SIvatW3s-tG53KtIlyEHRGg$) for guidelines on how to de-identify and prepare clinical data for publication. For a list of acceptable repositories, please see [http://journals.plos.org/plosone/s/data-availability#loc-recommended-repositories](https://urldefense.com/v3/__http:/journals.plos.org/plosone/s/data-availability*loc-recommended-repositories__;Iw!!JQ5agg!J26tD8jOp8XofgXZtoLkz1ia9DJnQaKXKBap8EponUvoq88SIvatW3s-tG53KtJea7S7Tw$).    We will update your Data Availability statement on your behalf to reflect the information you provide. | Yes Restriction  The use of such database is governed by a local ethic committee (named CADS-APHM) in accordance with the RGPD rules.  Each request must be submitted to: [cads@ap-hm.fr](mailto:cads@ap-hm.fr) and dpo@ap-hm.fr | Not applicable |
| 4. We note that you have included the phrase “data not shown” in your manuscript. Unfortunately, this does not meet our data sharing requirements. PLOS does not permit references to inaccessible data. We require that authors provide all relevant data within the paper, Supporting Information files, or in an acceptable, public repository. Please add a citation to support this phrase or upload the data that corresponds with these findings to a stable repository (such as Figshare or Dryad) and provide and URLs, DOIs, or accession numbers that may be used to access these data. Or, if the data are not a core part of the research being presented in your study, we ask that you remove the phrase that refers to these data. | We removed this sentence and gave the results. | Not applicable |
| 5. Please include captions for your Supporting Information files at the end of your manuscript, and update any in-text citations to match accordingly. Please see our Supporting Information guidelines for more information: [http://journals.plos.org/plosone/s/supporting-information](https://urldefense.com/v3/__http:/journals.plos.org/plosone/s/supporting-information__;!!JQ5agg!J26tD8jOp8XofgXZtoLkz1ia9DJnQaKXKBap8EponUvoq88SIvatW3s-tG53KtJ4lUockw$) | A caption is now included for supplementary file | Page 21 |

Reviewer 1

| **Reviewer comments** | **Author response and changes made** | **Page number** |
| --- | --- | --- |
| 1. This study evaluates the correlation between zip-code level neighborhood socioeconomic status (SES) and the risk of large for gestational age (LGA), focusing on newborn hospital birth admission claims in an urban area – Marseille – extracted from nationally representative administrative data called the French National Uniform Hospital Discharge Database. Statistical strategy is straightforward, applying multivariate logistic regression model. The result shows that lower zip-code level of neighborhood SES tend to be associated with LGA significantly, after adjusting for mother’s age, diabetes, obesity, smoking behavior and multiparity. Further, supposed that mother’s age is a proxy of duration of residency, the author(s) discuss that older mothers living in the area with lower SES might experience a cumulative disease risk over time and therefore, the intensification of the correlation between neighborhood SES and LGA increases along with mother’s age.  I fully agreed with the contributions and novelties highlighted by the author(s), such that this study sheds a light on the issue under the universal maternity coverage in a developed country – France – other than Northern American countries; and also it could provide clinicians additional information to help them understand mothers’ risks of LGA births, along with conventional ones, such as maternal diabetes, obesity and age. However, I think that the author(s) had better to explain carefully about the situation in France as a background to persuade international readers. I would like to provide both major and minor comments as follows. Hopefully, they are helpful for the author(s) to improve this paper. | We thank the Reviewer for her/his constructive analysis and we hope that our changes improved our manuscript. | Not applicable |
| 2. The current title “The role of neighborhood socioeconomic status in large for gestational age: a nationwide study” is unclear about target population to be analyzed. Further, although this study focused only on an urban area in an urban area – Marseille –, the subtitle (“a nationwide study”) might mislead the readers. So, I strongly suggest the author(s) to revised the title to the one which reflects the contents of this study more precisely. | We thank the Reviewer for this important comment and the title was changed as follows: “The role of neighborhood socioeconomic status in large for gestational age~~: a nationwide study~~” | Page 1 |
| 3. More detailed explanation may be helpful for international readers to understand the situation in France as a background information In the section of “BACKGROUND”, the author(s) had better to provide more detailed explanation how serious LGA births has been becoming, not only in France but also in other developed countries, showing some basic statistics (time-trend LGA births, if possible). Also, the author(s) emphasizes high standards of maternity care in France due to guaranteed access to care regardless of costs for pregnant women in 14th line of page 3, without any reference. So, the author(s) need to show some reference for this. Then, the author(s) had better to show basic statistics and/or to conduct institutional explanation for proving how high the standards of maternity care in France are, compared to other developed countries like OECD countries. Adding such more detailed clarifications may help to persuade international readers how important this study is. | As suggested by the Reviewer, we completed the introduction with some basic statistics and time-trend. We added the following sentence and the following references:  “Large for gestational age (LGA) is a growing public health problem in developed countries [1-3]. The prevalence of infants born LGA varies from 5 to 20% in developed countries (Henriksen, 2008). Recent studies suggest an increase in the number of LGA over the last two decades (Chiavaroli, 2016; Surkan, 2004). The number of LGA births is also expected to rise in the current epidemic context of obesity and diabetes, two major LGA risk factors [4]. In women with these metabolic complications, the risk of LGA increases twofold to threefold (Ehrenberg HM et al.2004; Black MH 2013).”  Henriksen T. The macrosomic fetus: a challenge in current obstetrics. Acta Obstet Gynecol Scand. 2008;87(2):134–45. doi: 10.1080/00016340801899289.  [Chiavaroli V](https://www.ncbi.nlm.nih.gov/pubmed/?term=Chiavaroli%20V%5BAuthor%5D&cauthor=true&cauthor_uid=27117061), [Castorani V](https://www.ncbi.nlm.nih.gov/pubmed/?term=Castorani%20V%5BAuthor%5D&cauthor=true&cauthor_uid=27117061), [Guidone P](https://www.ncbi.nlm.nih.gov/pubmed/?term=Guidone%20P%5BAuthor%5D&cauthor=true&cauthor_uid=27117061), [Derraik JG](https://www.ncbi.nlm.nih.gov/pubmed/?term=Derraik%20JG%5BAuthor%5D&cauthor=true&cauthor_uid=27117061), [Liberati M](https://www.ncbi.nlm.nih.gov/pubmed/?term=Liberati%20M%5BAuthor%5D&cauthor=true&cauthor_uid=27117061), [Chiarelli F](https://www.ncbi.nlm.nih.gov/pubmed/?term=Chiarelli%20F%5BAuthor%5D&cauthor=true&cauthor_uid=27117061), [Mohn A](https://www.ncbi.nlm.nih.gov/pubmed/?term=Mohn%20A%5BAuthor%5D&cauthor=true&cauthor_uid=27117061). Incidence of infants born small- and large-for-gestational-age in an Italian cohort over a 20-year period and associated risk factors. [Ital J Pediatr.](https://www.ncbi.nlm.nih.gov/pubmed/27117061) 2016 Apr 26;42:42. doi: 10.1186/s13052-016-0254-7.  Surkan PJ, Hsieh CC, Johansson AL, Dickman PW, Cnattingius S. Reasons for increasing trends in large for gestational age births. Obstet Gynecol. 2004;104(4):720-6. doi: 10.1097/01.AOG.0000141442.59573.cd. PubMed PMID: 15458892.  [Ehrenberg HM](https://www.ncbi.nlm.nih.gov/pubmed/?term=Ehrenberg%20HM%5BAuthor%5D&cauthor=true&cauthor_uid=15467573), [Mercer BM](https://www.ncbi.nlm.nih.gov/pubmed/?term=Mercer%20BM%5BAuthor%5D&cauthor=true&cauthor_uid=15467573), [Catalano PM](https://www.ncbi.nlm.nih.gov/pubmed/?term=Catalano%20PM%5BAuthor%5D&cauthor=true&cauthor_uid=15467573).The influence of obesity and diabetes on the prevalence of macrosomia. [Am J Obstet Gynecol.](https://www.ncbi.nlm.nih.gov/pubmed/?term=Ehrenberg+HM+et+al.2004+lga) 2004 Sep;191(3):964-8.  [Black MH](https://www.ncbi.nlm.nih.gov/pubmed/?term=Black%20MH%5BAuthor%5D&cauthor=true&cauthor_uid=22891256), [Sacks DA](https://www.ncbi.nlm.nih.gov/pubmed/?term=Sacks%20DA%5BAuthor%5D&cauthor=true&cauthor_uid=22891256), [Xiang AH](https://www.ncbi.nlm.nih.gov/pubmed/?term=Xiang%20AH%5BAuthor%5D&cauthor=true&cauthor_uid=22891256), [Lawrence JM](https://www.ncbi.nlm.nih.gov/pubmed/?term=Lawrence%20JM%5BAuthor%5D&cauthor=true&cauthor_uid=22891256).  The relative contribution of prepregnancy overweight and obesity, gestational weight gain, and IADPSG-defined gestational diabetes mellitus to fetal overgrowth. [Diabetes Care.](https://www.ncbi.nlm.nih.gov/pubmed/?term=Black+MH+2013+lga) 2013 Jan;36(1):56-62.  Concerning standards of care in France, we propose adding the following information and references:  “Exploring this issue in other countries and health care systems may provide additional information. France may offer an interesting view as the French Health system offers universal health coverage guaranteeing access to care regardless of cost for pregnant women[21]. Standards of maternity care are considered high, with a total of 7 recommended prenatal examinations. According the World Health Organization, prenatal examinations provide an important opportunity to prevent and manage concurrent diseases through integrated service delivery (WHO, 2016) and France is one of the European countries with the lowest percentage of late prenatal examinations (Wildman, 2003; Euro-Peristat, 2010). All medical costs during the perinatal period are covered at a rate of 100% by the French social security system. Investigating the role of SES on LGA at the whole population level with universal maternity coverage would therefore add a significant step in the identification of LGA risk factors.”  Integrated Management of Pregnancy and Childbirth (IMPAC). In: Maternal, newborn, child and adolescent health [WHO web page]. Geneva: World Health Organization (http://www.who.int/maternal_child_ adolescent/topics/maternal/impac/en/, accessed 17 October 2016).  Wildman K, Blondel B, Nijhuis J, Defoort P, Bakoula C. European indicators of health care during pregnancy, delivery and the postpartum period. Eur J Obstet Gynecol Reprod Biol. 2003 Nov 28;111 Suppl 1:S53-65.  EUROPEAN PERINATAL HEALTH REPORT Health and Care of Pregnant Women and Babies in Europe in 2010.  <https://www.europeristat.com/images/doc/Peristat%202013%20V2.pdf> | Page 3 |
| 4. Why Marseille? As long as I noticed, in the section of “METHODOLOGY”, there are no explanations why the author(s) chose Marseille from a nationwide population-based database in France. Then, from the 2nd bottom line in page 9 through page 10, the author(s) briefly mentioned why they chose an urban large city for this study, such that “Lastly, the accuracy of geographical methods based on residential postal code can nevertheless be put into question in studies including urban and rural populations with nonhomogeneous social neighborhood characteristics. This study avoided such a bias by limiting investigations to a single large city”. The author(s) definitely should reallocate this sentence to the “METHODOLOGY” section. Further, the author(s) should clarify the reason why they chose Marseille among multiple urban areas/cities in France and they have to discuss the possible sampling bias issues on the estimation caused by focusing on a large city like Marseille in “DISCUSSION” section. | As proposed by the Reviewer, we completed the methodology section as follows:  “All singleton infants whose parents were living in the city of Marseilles, France, between 2013 and 2016 were eligible for the study regardless of the maternity ward address. This study was part of a regional project aimed to identify factors influencing perinatal health outcomes. Marseilles is the second-largest city of France, with almost 860,000 inhabitants and 13,000 births per year and is one of the French cities with the highest levels of social inequality (OCDE, 2013). The size of the city was enough to use geographic socio-economic index based on zip-codes. Previous works in Marseilles reported that social inequalities were well captured by zip-codes, *i.e.,* inhabitants shared resembling socio-economics characteristics in a same zip-code (Bocquier, 2013), which is not demonstrated in other French cities and in studies associating urban and rural populations. “  Bocquier A, Cortaredona S, Boutin C, David A, Bigot A, Chaix B, Gaudart J, Verger P. Small-area analysis of social inequalities in residential exposure to road traffic noise in Marseilles, France. Eur J Public Health. 2013 Aug;23(4):540-6.  OCDE. Towards more inclusive growth in the metropolitan area of Aix-Marseille: International insights. 2013. <https://www.oecd.org/cfe/regional-policy/Summary-Aix-Marseille.pdf>.  In the discussion, we completed the limitations as follows:  “However, conducting a study in a single French city may limit the generalization of our findings. Future studies should thus confirm our findings in other geographical areas” | Pages 4 and 11 |
| 5. A flowchart for the procedure of extracting the data to be analyzed is necessary In the first paragraph of “RESULTS” section in page 6, the author(s) describe how to extract the data to be analyzed in this study. I suggest to bring this explanation to “METHODOLOGY” section and show it by a figure of flowchart. It may be very helpful for the readers to understand how the data is constructed. | We thank the reviewer for this helpful suggestion.  We added a flowchart and an explanation in the method section.    “From the PMSI database, we selected all in-hospital stays corresponding to a neonatal diagnosis-related group (DRG n°15 in France and with an age equal to 0 day) and with an address codes localized in Marseilles. We excluded in-hospital stays with linking problems, transfers from hospitals in another geographical area, infants issued from multiple pregnancies or when the number of infants was not specified, and infants with small for gestational age (birth weights lower than the 10th percentile). Then, we defined two populations: cases were infants with LGA (birth weights lower than the 10th percentile) and controls were infants with AGA.” | Figure 2 and  Page 4 |
| 6. Justification for the threshold “p-values<0.2” In the 7-9th line of page 5, the author(s) said “Variables relevant to the model were selected based on a threshold p<0.2”. Please provide the rationale for choosing this threshold. Otherwise, the selection of explanatory variables seems to be with some ‘intention’. If the author(s) do not have any clear justifications for this, they should to some stepwise logistic regression analyses. | As suggested by the reviewers, we applied stepwise logistic regression for the different analyses. The findings are updated in the tables (without changes in significance) and in the method section.  “To determine whether the NDI was an independent factor associated with LGA, we performed multivariate stepwise backward regression analyses.” | Page 6 |
| 7. Sensitivity analysis excluding/imputing missing data on maternal age From the bottom line of page 5 through page 6, the author(s) mentioned the above sensitivity analyses. However, I do not see any results for this. Please show the results of sensitivity analysis, at least in supplementary tables. Also, if the author(s) did some imputation for missing maternal age, they should briefly explain which imputation method they used, either in the main body of text or in the appendix | The sensitivity analyses are now presented in table 3.  The imputation is also better explained in the method section: “Maternal age being absent for 10% of women, we performed an analysis with imputation to the mean (i.e., replacement of maternal age by the mean age of mothers who delivered in the same maternity ward in the same year) and an analysis excluding mothers with missing data.” | Page 6 |
| 8. Test statistics for regression analyses are necessary to be shown in Table 3 Test statistics for validity of the model should be shown, such as log likelihood, pseudo-R2 values, and Akaike's Information Criterion (AIC). | As suggested, we added test statistics in table 3 such AIC. | Table 3 |
| 9. Univariate results are not necessary to be shown in Table 3 In Table 3, the author(s) show both results of univariate and multivariate logistic regression analyses. However, I do not see any meaning to show univariate results (besides, the results do not seem to be different much). Rather, it is more interesting to show the results of supplementary tables 2 and 3. Besides, in page 7, the author(s) explain the results (ORs) of these supplementary tables in detail. For example, the author(s) said “The strength of the association between SES and LGA increased with maternal age, reaching an aOR of 1.59 (95% CI: 1.26; 1.78 p<0.0001)...” , which cannot be seen in any tables in the main body of manuscript. | We replaced univariate results by multivariate results for each analysis in Table 3.  See Table 3 and the method/result sections:  “All the multivariate analyses are presented in table 3.”  The strength of the interaction was now presented in the footnotes of the table 3 and in the text. | Page 7 |
| 10. How to estimate the results for Figure 1 & supplementary table 3 I do not see how to extract the results of Figure 1 (supplementary table 3). I guess the author(s) introduce interactive terms of maternal age and NDI levels into logistic regression analysis. If possible, please clarify the estimation model in “METHODOLOGY” section. Otherwise, the reader must be lost in a series of explanation about the results of Figure 1 in page 7 | The method section is now completed, explaining that the interactions were tested and for reasons of readability, it is presented in the form of a figure.  “Interaction effects were tested between the different variables, in particular the interaction between maternal age and NDI levels presented in the figure 1”. | Page 6 |
| 11. In page 4, the translation of the name of dataset sounds a little strange. How about “the French National Uniform Hospital Discharge Data” or “the French National Uniform Hospital Discharge Database”. I do not think that the author(s) need to repeat “Data Set Database”. | Corrected as follows:  “This population-based cohort study was based on newborn hospital birth admission charts from the French National Uniform Hospital Discharge Database (Programme de Médicalisation du Système d’Information, PMSI), which systematically collects administrative and medical information, including obstetrical characteristics.” | Page 4 |
| 12. In the 8th line of page 5, what is (Audipog) (19)? Please clarify. | We removed Audipog which is in fact not necessary. The reference 19 is sufficient. | Page 5 |
| 13. In “RESULTS” in page 6, the author(s) said “The rates of infants born to mothers with advanced age (more than 35 years)” is “51%”, which is different from the value (“20.1% for age 35 and older”) shown in Table 1. | We are sorry for the mistake; we corrected it.  “The rates of infants born to mothers with advanced age (i.e., more than 35 years), GDM and preeclampsia were 20.1%, 6.4% and 1.8%, respectively.” | Page 7 |
| 14. Same as (1), In “RESULTS-LGA population characteristics”, the author(s) said “In comparison with AGA infants, LGA infants were more likely to be born to older (maternal age more than 35 years) and multiparous mothers (p<0.0001)”. However, Table 1 shows an opposite result, such that the ratio of Cesarean delivery tends to be significantly larger for LGA infants so that LGA infants were less likely to be born to multiparous mothers. | We are sorry for this misunderstanding.  Multiparity can only be computed on vaginal deliveries. Thus the percentages of multiparity which are presented in table 1 excludes caesarian deliveries. That’s why we have performed sensitivity analysis on this subpopulation (vaginal deliveries) only when taking into account multiparity in the model.  “*Multiparity was calculated for infants delivered vaginally (data not available for cesarean delivery).” | Table 1. |
| 15. At the second line from the bottom of page 6, the author(s) explains about GA (p=0.81). Then, again, the author mentioned about GA at the first line of page 7, such as “While the mean GA and preterm birth rate were comparable”. Better to erase either one. | We removed the following sentence: “The mean LGA birth weight was significantly higher (p<0.001) for a similar GA (p = 0.81).” |  |
| 16. At the forth line in page 7, the author(s) mentioned “with higher rates of hypoglycemia, hypocalcemia”. Yet, Table 1 does not show the rates of these variables. Please add these in Table 1, if the author(s) use these variables for the further statistical analyses. | We add it in table 1: “***metabolic disorders: hypoglycemia and hypocalcemia”. |  |
| 17. In “RESULTS-Neighborhood deprivation and perinatal outcomes” in page 7, the author(s) mentioned “… and to be prenatally exposed to maternal smoking”, which is different from the results shown in Table 2. Table 2 shows that infants born to mothers living in Q2 and Q3 of NDI are more likely to expose to maternal smoking (1.9%), than Q4 (1.4%) and Q1 (0.8%). Please revise the sentence | We have now better explained this sentence: “Infants from NDI levels 2, 3 and 4 were more frequently exposed to maternal smoking than those from NDI level 1 (p < 0.001)”. | Page 7 |
| 18. Again, in the same section as above (5), the author(s) repeatedly explain about the ratio of LGA. In the 2-3 lines of this section, the author(s) said “The proportion of LGA varies by NDI levels, reaching 12% in NDI level 4”. Then, in the 3-4th lines from the bottom of this section, the author(s) repeatedly explain “…, while we observe a higher rate of LGA with the NDI (12.3% in NDI level 4 vs 9.9% in NDI level 1, p<0.0001)”. Please drop the first part. | As suggested by the Reviewer, we removed the following sentence:  “~~while we observed a higher rate of LGA with the NDI (12.3% in NDI level 4 vs 9.9% in NDI level 1, p <0.0001).~~” | Page 7 |
| 19. As mentioned in my major comments, I do not see any reasons why the author(s) show “Univariate analysis” in Table 3, which is not necessary. Instead, the author(s) should show the results of supplemental table 2 & supplemental data table 3. | Multivariate analyses are now in the table 3 and supplementary files 2 and 3 were removed. | Table 3 |

Reviewer 2

| **Reviewer comments** | **Author response and changes made** | **Page number** |
| --- | --- | --- |
| 1. This manuscript examined associations between neighbourhood-level socioeconomic status and large-for-gestational age (LGA) risk in a sample of 43,309 births between 2013 and 2016 in Marseilles, France. Independent of other variables, lower neighbourhood socioeconomic status was associated with increased risk for LGA status, and this trend was pronounced for older women. This is a well written and interesting article. | We thank the Reviewer for her/his constructive analysis and we hope that our changes improved our manuscript. | Not applicable |
| 2. First, exploring interactions between neighbourhood SES and other variables, and specifically with age, were not developed or justified in the Introduction. Theoretical background justifying the examination of this interaction, and preparing the reader for presentation of interaction findings in the Results, is needed. | We completed the introduction with this study reporting that older women are at increased risk of large for gestational age.  “LGA may also be influence by maternal age: advanced maternal age (i.e., women older than 35) have been suggested to increase the risk of LGA (Kenny LC). Birth to advanced maternal age have increased over the past decades reaching about 10 % to 20 % in high income countries (Breart, 2003 ; Martin, 2018 ; Eurostat). These older women are at increased of various complications during pregnancy such as preeclampsia, GDM, low birth weight and perinatal mortality [30-32]. A large body of literature has reported the impact of neighborhood socioeconomic status (SES) on perinatal and birth outcomes, including low birth weight, small for gestational age and preterm birth[14-16]. However, the influence of neighborhood SES on LGA and combined effects with other LGA factors including maternal age has received little attention.”  Kenny LC, Lavender T, McNamee R, O'Neill SM, Mills T, Khashan AS. Advanced maternal age and adverse pregnancy outcome: evidence from a large contemporary cohort. PLoS One. 2013;8(2):e56583.  Breart G, Barros H, Wagener Y, Prati S. Characteristics of the childbearing population in Europe. Eur J Obstet Gynecol Reprod Biol. 2003;111(Suppl 1):S45–S52;  Martin JA, Hamilton BE, Osterman MJK, Driscoll AK, Drake P. Births: Final Data for 2017. Natl Vital Stat Rep. 2018 Nov;67(8):1-50.  European Commission: eurostat. Fertility statistics (<http://epp.eurostat.ec.europa.eu/statistics_explained/index.php/Fertility_statistics>, accessed on 20/01/2013 | Page 3 |
| 3. Related, the authors note in the Results that “as expected, maternal smoking was a protective factor.” For the reader, this finding is not expected at all because the authors provide no literature discussing the effect of smoking on LGA risk. And smoking during pregnancy is not usually considered a good or “protective” factor, which is what this sentence implies. Please consider revising and providing more context for this finding | We agree with the reviewer that the sentence is awkward. We removed as expected, we insisted on the negative impact of smoking. The sentence was changed as following:  “~~As expected,~~ Maternal smoking was at lower odds of LGA (p<0.001).” | Page 8 |
| 4. Second, did the authors consider excluding children with congenital defects, which could have affected pregnancy and infant size at birth? | We agree with the Reviewer that this data was not considered in our work and we added this issue in the limitation section:  “The French National Uniform Hospital Discharge Data Set Database does not include some relevant individual factors, including educational status, ethnicity, maternal BMI before or in early pregnancy, maternal body weight gain, hyperglycemic oral test, maternal nutritional status and data regarding fetal growth. Thus, we were unable to identify how these factors and their severity may mediate the effects of neighborhood deprivation on LGA. Some comorbidities and clinical characteristics derived from ICD codes could be underreported, such as maternal smoking or obesity. In addition, we did not analyzed congenital defects which could affect pregnancy and infant size at birth. Specific studies should explore this issue. Such limitations may nevertheless be mitigated by the fact that our study is the first large population-based study performed in industrialized countries. Our data were extracted from the French national database, which accurately registers hospitalized newborns, maternal health data and the domicile postal code of the mother’s address. All consecutive deliveries in private or public hospitals providing different levels of health care were collected in this registered database system, which strengthens the findings of the study.” | Page 10 |
| 5. Third, a key and interesting finding is that the effect of neighbourhood socioeconomic status on LGA status is larger for older women compared to younger. The authors also report, however, that older women in the more- and less-deprived neighbourhoods seem to differ in significant ways. Could the authors explicitly characterize the older women in more-deprived neighbourhoods, provide context for those differences, and tie that into clinical implications for these findings? | We agree with the Reviewer that this is an important issue that we should develop. We propose completing the following section as follows:  “The underlying mechanism is unclear. Although we do not have information on the duration of residence, it may be reasonably hypothesized that LGA risk may increase with the duration of the abovementioned SES-associated factor exposure. This hypothesis could be supported by previous studies reporting life-course accumulation of neighborhood disadvantage and its impact on health[34]. It can be speculated that older mothers with low SES experience a cumulative disease risk over time, including repeated stressful adversity events, diabetes, obesity and multiparity, which in turn promote LGA[3]. Older mothers with low SES have been described to be associated with several risky behavioral factors such as psychosocial issues, smoking practices, alcohol exposure, stress, which should be specifically targeted early in pregnancy (Delpisheh, 2006, 2008; Jacobsson, 2004). If this cumulative risk hypothesis was confirmed, our results would suggest that the preventative efforts in current and individual conditions are not sufficient and that early health and social support (individual and/or collective) interventions are also necessary to improve perinatal and birth outcomes.”  Delpisheh A. Brabin L. Brabin BJ. Pregnancy, smoking and birth outcomes. Womens Health. 2006;2:389–404.  Delpisheh A, Brabin L, Attia E, Brabin BJ. Pregnancy late in life: a hospital-based study of birth outcomes.J Womens Health (Larchmt). 2008 Jul-Aug;17(6):965-70.  Jacobsson B. Ladfors L. Milsom I. Advanced maternal age and adverse perinatal outcome. Obstet Gynecol. 2004;104:727–733. | Page 10 |
| 6. Fourth, the authors say in the Discussion that “neighbourhood SES may thus be a good proxy for capturing other mechanisms associated with LGA.” The proxies listed are very heterogenous, and as the authors note not well captured in this data set. I would advise caution in making such a statement about using neighbourhood socioeconomic status as a “proxy” for everything from poor health behaviours, endocrine disruptors and race/ethnicity. Please revise. | We agree with the Review and we changed the sentence as follows:  “Neighborhood SES may ~~thus~~ be a good proxy to capture other mechanisms associated with LGA such as poor health behaviours, endocrine disruptors and race/ethnicity characteristics. Future studies should explore if neighborhood SES validly capture characteristics such as inadequate diet behaviors (e.g., micronutrient and vitamin deficiencies), endocrinal disruptors exposure and ethnic characteristics.” | Page 9 |
