# Supplementary material for: The role of neighbourhood socioeconomic status in large for gestational age
Source: PLoS One. 2020 Jun 5;15(6):e0233416. doi: 10.1371/journal.pone.0233416 (PMC7274403; doi:10.1371/journal.pone.0233416)
Supplement: Attachment — Submitted filename: Response to reviewers_Letter_02052020.docx [file pone.0233416.s004.docx]

Editor

| Editor comments | **Author response and changes made** |
| --- | --- |
| Firstly you say in the Discussion that “neighbourhood SES may thus be a good proxy for capturing other mechanisms associated with LGA.” The proxies listed are very heterogenous, and as noted not well captured in this data set. I would advise caution in making such a statement about using neighbourhood socioeconomic status as a “proxy” for everything from poor health behaviours, endocrine disruptors and race/ethnicity. Please revise accordingly.” | As suggested by the Editor, we removed the notion of “proxy”, which is irrelevant. We prefer to talk about underlying mechanisms that may explain the SES and LGA association.  ~~Our findings Neighborhood SES may be a good proxy for capturing other~~ ~~mechanisms associated with LGA~~ The association between neighborhood SES and LGA may be explained by mechanisms, such as poor health behaviours, endocrine disruptors and race/ethnicity characteristics. Future studies should explore ~~if~~ the association between neighborhood SES ~~validly capture characteristics such as~~ and inadequate diet behaviors (e.g., micronutrient and vitamin deficiencies[41]), endocrine disruptor exposure and ethnic characteristics. |
| Secondly I am concerned about the following paragraph: “In line with the previous point, LGA prevalence in deprived neighborhoods was not expected to be so high (i.e., 25% higher than in less deprived neighborhoods) due to the universal health coverage that is supposed to reduce health care inequalities[27]. One explanation is that access to care is a multidimensional concept consisting of both financial and nonfinancial dimensions [43]. Our findings may suggest that nonfinancial barriers may substantially limit access to care despite universal health coverage in this study. In accordance with this hypothesis, a previous French study performed in Paris reported insufficient healthcare follow-up in women with low socioeconomic status[44]. Future studies should better explore nonfinancial barriers to care, and targeted interventions should be proposed for reducing nonfinancial barriers in these deprived neighborhoods.”  This paragraph makes it seem as if you are unfamiliar with the vast literature on disparities, which identify myriad factors, in addition to health coverage, that contribute to health and health care disparities. Also, LGA prevalence is a health disparity, not a health care disparity, so please consider the contributors to both and revise. | We agree with the Editor that this section is awkward. More simply, we would like to highlight in this section that universal health coverage is important but not sufficient to to oppose the myriad factors that contribute to health and health care disparities. Universal coverage still face unresolved challenges in France, such as persistent health inequities linked to social determinants of health. Even if these issues are well-known, underlying social determinants that drive persistent inequities including levels and distribution of income, education, housing, nutrition and safety should be better considered by health policies in France.  We propose the following changes:  “~~In line with the previous point,~~ LGA prevalence in deprived neighborhoods ~~was not expected to be so high (i.e.,~~ was 25% higher than in less deprived neighborhoods. ~~) due to the universal health coverage that is supposed to reduce health care inequalities[27]~~.  This finding confirm that LGA remains an important health disparity in France and that universal health coverage is not sufficient to oppose the multiple factors that contribute to health and health care disparities [43]: levels and distribution of income, education, housing, nutrition and safety [Howell, 2018; Warnecke, 2008].  ~~One explanation is that access to care is a multidimensional concept consisting of both financial and nonfinancial dimensions [43]. Our findings may suggest that nonfinancial barriers may substantially limit access to care despite universal health coverage in this study.~~ In accordance with this ~~hypothesis~~ finding, a previous French study performed in Paris reported insufficient healthcare follow-up in women with low socioeconomic status [44].  Future studies should better explore ~~nonfinancial barriers to care~~ economic, social, or environmental disadvantages, and targeted interventions should be proposed for reducing health disparities ~~nonfinancial barriers~~ in these deprived neighborhoods [Jones, 2019].”  We added the following three references on health disparities:  Elizabeth A Howell. Reducing Disparities in Severe Maternal Morbidity and Mortality. Clin Obstet Gynecol. 2018 Jun; 61(2): 387–399.  Warnecke RB, Oh A, Breen N, Gehlert S, Paskett E, Tucker KL, Lurie N, Rebbeck T, Goodwin J, Flack J, Srinivasan S, Kerner J, Heurtin-Roberts S, Abeles R, Tyson FL, Patmios G, Hiatt RA.  Approaching health disparities from a population perspective: the National Institutes of Health Centers for Population Health and Health Disparities.  Am J Public Health. 2008 Sep; 98(9):1608-15.  Nancy L Jones , Stephen E Gilman, Tina L Cheng, Stacy S Drury, Carl V Hill, Arline T Geronimus. Life Course Approaches to the Causes of Health Disparities. Am J Public Health 2019 Jan;109(S1):S48-S55. |
| You state “To our knowledge, only a few studies in the United States and Canada have addressed this issue with conflicting results [28-30].” It would be helpful to know what those conflicting results were, and if possible, why the authors believe these studies have produced conflicting results and, in the discussion, how this study addresses these conflicting results. | We propose to add this justification in the introduction:  “To our knowledge, only a few studies in the United States and Canada have addressed this issue with conflicting results, e.g., two studies with increased risk and one study with decreased risk of LGA in lower neighborhood SES [28-30]. Further studies are thus needed to confirm the influence of neighborhood SES on LGA.”  In the discussion,  “To date, the association between SES and LGA has rarely been investigated, especially in industrialized countries with developed health care systems. Our findings confirm the results of two previous studies [28,30] in another socio-economic and cultural context and with a large population but contradict the results of Shankardass et al [29] which did not adjust their findings on potential confounding characteristics such as in study.” |
| You should spell out abbreviations the first time they are used (e.g., GDM, AGA). They should consider using fewer abbreviations to improve readability, especially since this is geared to a multi-disciplinary audience who are not likely to be familiar with these abbreviations. | As suggested, we spelled out abbreviations the first time. We replaced GDM by gestational diabetes mellitus in the whole manuscript. |
| I recommend another round of editing, as there are some grammatical errors. | Manuscript edited by AJE, see new certificate |
